# Unraveling the function of *Arabidopsis thaliana* OS9 in the endoplasmic reticulum-associated degradation of glycoproteins

**DOI:** 10.1007/s11103-012-9891-4

**Published:** 2012-02-11

**Authors:** Silvia Hüttner, Christiane Veit, Jennifer Schoberer, Josephine Grass, Richard Strasser

**Affiliations:** 1 Department of Applied Genetics and Cell Biology, BOKU-University of Natural Resources and Life Sciences, Muthgasse 18, 1190 Vienna, Austria; 2 Department of Chemistry, BOKU-University of Natural Resources and Life Sciences, Muthgasse 18, 1190 Vienna, Austria; 3 Present Address: Department of Biological and Medical Sciences, Faculty of Health and Life Sciences, Oxford Brookes University, Gipsy Lane, Headington, Oxford, OX3 0BP UK

**Keywords:** Protein glycosylation, ERAD, Protein quality control, Posttranslational modification, Unfolded protein response, ER stress

## Abstract

In the endoplasmic reticulum, immature polypeptides coincide with terminally misfolded proteins. Consequently, cells need a well-balanced quality control system, which decides about the fate of individual proteins and maintains protein homeostasis. Misfolded and unassembled proteins are sent for destruction via the endoplasmic reticulum-associated degradation (ERAD) machinery to prevent the accumulation of potentially toxic protein aggregates. Here, we report the identification of *Arabidopsis thaliana* OS9 as a component of the plant ERAD pathway. OS9 is an ER-resident glycoprotein containing a mannose-6-phosphate receptor homology domain, which is also found in yeast and mammalian lectins involved in ERAD. OS9 fused to the C-terminal domain of YOS9 can complement the ERAD defect of the corresponding yeast Δ*yos9* mutant. An *A. thaliana* OS9 loss-of-function line suppresses the severe growth phenotype of the *bri1*-*5* and *bri1*-*9* mutant plants, which harbour mutated forms of the brassinosteroid receptor BRI1. Co-immunoprecipitation studies demonstrated that OS9 associates with Arabidopsis SEL1L/HRD3, which is part of the plant ERAD complex and with the ERAD substrates BRI1-5 and BRI1-9, but only the binding to BRI1-5 occurs in a glycan-dependent way. OS9-deficiency results in activation of the unfolded protein response and reduces salt tolerance, highlighting the role of OS9 during ER stress. We propose that OS9 is a component of the plant ERAD machinery and may act specifically in the glycoprotein degradation pathway.

**Electronic supplementary material:**

The online version of this article (doi:10.1007/s11103-012-9891-4) contains supplementary material, which is available to authorized users.

## Introduction

In all eukaryotes, the endoplasmic reticulum (ER) is the site of folding, maturation and assembly of proteins entering the secretory pathway. In the ER these soluble and membrane-bound proteins attain their native conformation before they are delivered to different compartments of the endomembrane system and to the extracellular space. Folding and maturation of the proteins is guided by ER-resident chaperones, lectins and enzymes that work together in a protein quality control system to ensure that only correctly folded and assembled proteins are released to the Golgi (Ellgaard and Helenius 2003). To maintain the protein homeostasis in the ER terminally misfolded proteins or proteins that do not integrate into cognate complexes are subjected to degradation in a tightly regulated multistep process referred to as ER-associated degradation (ERAD) (McCracken and Brodsky 1996). In this process non-native proteins are recognized, retrotranslocated to the cytosol, ubiquitinated and finally destroyed by the 26S proteasome (Vembar and Brodsky 2008).

Many proteins of the secretory pathway are modified with N-linked oligosaccharides, which are added mainly co-translationally upon entry of the nascent polypeptide chain into the ER. The attached N-glycans can have a direct impact on the folding process of proteins or they indirectly affect folding by subjecting polypeptides to the calnexin/calreticulin cycle (Helenius and Aebi 2001). Moreover, specific N-glycan structures on aberrant proteins play a crucial role for ERAD. For yeast it has been demonstrated that the Man_7_GlcNAc_2_ oligosaccharide, which is generated by the subsequent action of two class I α-mannosidases, exposes a terminal α1,6-linked mannosyl-residue that marks glycoproteins for degradation by ERAD (Clerc et al. 2009). This specific mannose residue is recognized by the ER-resident lectin YOS9, which acts as a quality control receptor and links the recognition of glycoproteins destined for degradation to the ubiquitin-proteasome system (Quan et al. 2008; Clerc et al. 2009).

YOS9 and its mammalian homologs OS-9 and XTP3-B contain mannose-6-phosphate receptor homology domains (MRH) and mutations in the conserved carbohydrate-binding sites reduce the rate of misfolded glycoprotein breakdown (Bhamidipati et al. 2005; Szathmary et al. 2005; Hosokawa et al. 2009). In the current model for yeast ERAD, YOS9 interacts with the ER membrane-embedded HRD3-HRD1 ubiquitin ligase complex. The luminal domain of HRD3 recognizes misfolded polypeptide segments of aberrant proteins and YOS9 scans them for the presence of the glycan signal, leading to retrotranslocation of the ERAD substrates to the cytoplasm, polyubiquitinylation by the HRD1 E3 ubiquitin-ligase and proteasomal degradation. Analogous to this model, the mammalian OS-9 and XTP3-B proteins interact with the SEL1L-HRD1 complex and play a crucial role for ERAD of glycoproteins. In contrast to the well-characterized yeast and mammalian ERAD pathways comparatively little is known about the process in plants, especially the glycan-dependent steps remain elusive (Vitale and Boston 2008). Earlier studies reported on the degradation of non-glycosylated proteins (Brandizzi et al. 2003; Müller et al. 2005) or on glycoproteins, where the glycosylation status did not play a specific role for the degradation of the substrates (Marshall et al. 2008). The first indication that glycan trimming reactions are involved in the degradation of misfolded proteins in plants came from the analysis of defective forms of the Arabidopsis brassinosteroid receptor BRI1 (Hong et al. 2008, 2009). BRI1 is a plasma membrane located leucine-rich repeat receptor like kinase that contains 14 putative N-glycosylation sites in its ectodomain (Li and Chory 1997). The mutant BRI1 forms BRI1-9 (S662F) and BRI1-5 (C69Y) were found to be retained in the ER by quality control mechanisms. Treatment with the specific class I α-mannosidase inhibitor kifunensine (Elbein et al. 1990) stabilized the mutated BRI1 forms and suppressed the characteristic growth phenotype of the *bri1*-*5* and *bri1*-*9* mutants indicating that BRI1-5 and BRI1-9 are subjected to ERAD (Jin et al. 2007; Hong et al. 2008, 2009). In Arabidopsis kifunensine specifically inhibits α-mannosidases (MNS proteins) involved in N-glycan trimming (Liebminger et al. 2009) and mutants which are deficient in the ALG12 α1,6-mannosyltransferase and lack the specific α1,6-mannosyl residue are defective in degradation of mutated BRI1-5 and BRI1-9 (Hong et al. 2009). These results highlight that a similar glycan-dependent ERAD pathway is functional in plants, which involves removal of mannose residues from distinct oligosaccharides and recognition of a specific glycan signal by an ER-resident lectin similar to YOS9 and OS-9/XTP3-B.

To identify proteins involved in ERAD of plant glycoproteins we analyzed an Arabidopsis protein with a MRH-domain for its role in the degradation of the glycoprotein ERAD substrates BRI1-5 and BRI1-9. Here, we describe the involvement of Arabidopsis OS9 in ERAD and we show that OS9 deficiency activates the unfolded protein response (UPR) and results in increased susceptibility to salt stress.

## Materials and methods

### Plant materials

*Arabidopsis* *thaliana* Col-0 is the ecotype for all mutants, except for *bri1*-*6* (En-2 ecotype) and *bri1*-*9* (Ws-2 ecotype). All plants were grown under long-day conditions at 22°C as described previously (Liebminger et al. 2009). T-DNA insertion lines *os9*-*1* (SALK_029413), *sel1l* (SALK_109430), *bri1*-*6* and *bri1*-*5 bak1*-*1D* were obtained from the European Arabidopsis Stock Centre (http://arabidopsis.info). The *bri1*-*9* seeds were a kind gift of Frans E. Tax. To obtain the *bri1*-*5* mutant, *bri1*-*5 bak1*-*1D* was backcrossed to Col-0 and the absence of the T-DNA in the *BAK1* locus (Li et al. 2002) was confirmed by genotyping. For treatments with NaCl, KCl, tunicamycin (Sigma–Aldrich, http://www.sigmaaldrich.com) and paraquat (Sigma–Aldrich) seedlings were grown on Murashige and Skoog (MS) medium as indicated. *Nicotiana* *benthamiana* plants were grown under long-day condition at 24°C.

### PCR genotyping

For *os9*-*1*, the T-DNA insertion was confirmed by PCR using the left border primer LBa1 and the gene-specific primer At5g35080_1F and LBa1/At5g35080_2R (Table S1). Homozygous *os9*-*1* plants were identified by screening with primers At5g35080_1F/_2R and homozygous *sel1l* lines were identified with primers LBa1/At1g18260_1F and At1g18260_1F/_2R. *bri1*-*5*, *bri1*-*6* and *bri1*-*9* were crossed with *os9*-*1* and the double mutants were identified by PCR genotyping and DNA sequencing. To genotype *bri1*-*5*, a part of the *BRI1* locus was amplified with primers BRI1_15F/_16R. For confirmation of the *bri1*-*6* mutation, primers BRI1_10F/_14R were used for amplification, while for *bri1*-*9* primers BRI1_13F/_14R were used.

### Reverse transcription-PCR

RNA isolation and cDNA synthesis was done as described previously (Strasser et al. 2007). The *OS9* coding region including a part of the 5′- and 3′-untranslated regions was amplified using primers At5g35080_6F/_7R. To detect *OS9* transcripts, PCR was performed from cDNA using the primers At5g35080_3F/_2R.

### Plasmid construction and generation of transgenic plants

To create C-terminal fusion proteins, the *OS9* coding region was amplified from cDNA by PCR with primers At5g35080_4F/_5R and ligated into *Xba*I/*Bam*HI digested plasmids p20F (Strasser et al. 2007), p20F-Fc (Schoberer et al. 2009) and p31 (like p20F but mRFP instead of GFP) resulting in the binary plant expression vectors p20-AtOS9-GFP (35S:OS9-GFP), p20-AtOS9-Fc-GFP (35S:OS9-GFPglyc) and p31-AtOS9-mRFP (35S:OS9-mRFP), respectively. OS9 containing the R201A mutation was generated by site-directed mutagenesis of p20-AtOS9-Fc-GFP using the Quikchange II kit (Stratagene, http://genomics.agilent.com/) with the primers At5g35080_23F/_23R. For complementation of the *os9*-*1* mutant, wild type, *os9*-*1* and *os9*-*1 bri1*-*5* plants were floral dipped with *Agrobacterium tumefaciens* strain UIA143 containing p20-AtOS9-GFP, p20-AtOS9-Fc-GFP and p20-AtOS9R201A-Fc-GFP, respectively, and transgenic plants were selected on MS plates containing 50 μg mL^−1^ kanamycin.

For generation of transgenic plants expressing BRI1 with a C-terminal myc-tag, the BRI1 coding region was amplified from cDNA with primers BRI1_1F/_2R, *Bam*HI/*Nco*I-digested and ligated into *Bam*HI-linearized pPT8 (Strasser et al. 2006) to create the binary plant expression vector pPT8-BRI1-myc. The vector pPT8-bri1-9-*myc* was created by site-directed mutagenesis using primers BRI1_7F/_7R. pPT8-bri1-5-myc was created by swapping the N-terminal part of BRI1 in pPT8-BRI1-myc with the N-terminal part of bri1-5 (obtained by amplification with primers BRI1_17F/_18R) by making use of the internal *Xba*I restriction site. BRI1-GFP and BRI1-5-GFP were generated in the same way by cloning of the BRI1 fragments into p20F. The plasmid expressing a C-terminal HA-tagged SEL1L (p60SEL1L) was generated by amplification of the *SEL1L* open reading frame with primers At1g18260_10F/_11R. The product was subcloned, *Spe*I/*Bgl*II-digested and ligated into *Xba*I/*Bam*HI-linearized p60 plasmid, which is a derivative of p20F that contains a 3× HA-tag sequence instead of GFP.

### Subcellular localization of OS9-GFP

Transient expression in leaves of 4–5 week-old *N. benthamiana* plants was done by infiltration of a suspension of the *A. tumefaciens* strain UIA143 (OD_600_ of 0.1–0.2) containing p20-AtOS9-GFP, p20-AtOS9-Fc-GFP or p31-AtOS9-mRFP. Sections of infiltrated leaves were analysed 1–2 days after infiltration on a Leica TCS SP2 confocal microscope as described recently (Schoberer et al. 2009). For co-localization, agrobacteria containing the ER/Golgi-marker GnTI-CaaaTS-mRFP (Schoberer et al. 2009) were co-infiltrated with p20-AtOS9-GFP or p20-AtOS9-Fc-GFP.

### Co-purification

For co-purification experiments, leaves of 4–5 week-old *N. benthamiana* plants were co-infiltrated with agrobacteria (OD_600_ of 0.2–0.3) containing p20-AtOS9-Fc-GFP or p20-AtOS9R201A-Fc-GFP (both contain a domain from the IgG heavy chain which binds very efficiently to Protein A) and either pPT8-BRI1-myc, pPT8-bri1-5-myc, pPT8-bri1-9-myc or p60SEL1L. Proteins were extracted from 1 g of leaves with RIPA buffer (Sigma–Aldrich) supplemented with 1% (v/v) protease inhibitor cocktail (Sigma–Aldrich), followed by incubation on ice for 30 min and centrifugation at 10,000*g* for 10 min at 4°C. The cleared supernatant was incubated with 40 μL rProtein A-Sepharose (GE Healthcare, http://www.gelifesciences.com) for 1 h at 4°C on a rocking shaker and washed extensively with RIPA buffer on a Micro Bio-Spin Chromatography column (Bio-Rad, http://www.bio-rad.com). Finally, the protein fractions were eluted by boiling in 1× SDS sample buffer, separated by 7% SDS–PAGE and analyzed by immunoblotting using rabbit polyclonal anti-myc (A-14, Santa Curz Biotechnology, http://www.scbt.com), mouse monoclonal anti-HA (12CA5) or anti-BRI1 (custom-made in rabbits against the peptide: CGKRPTDSPDFGDNN, GenScript, http://www.genscript.com/) and anti-OS9 (custom-made in rabbits against the peptide: LPEDSPFHPGDNLEC, GenScript) antibodies.

### Endo H treatment

Leaves from 4 to 6 week-old plants were frozen in liquid nitrogen, homogenized in a mixer mill and a crude protein extract obtained by adding 1.5× SDS sample buffer, followed by incubation on ice for 30 min. After centrifugation at 10,000*g* for 10 min at 4°C, the supernatant was denatured at 95°C for 10 min in 1× Denaturing Buffer (NEB, http://www.neb-online.de) and incubated with or without 1,000 U of Endo H (NEB) in 1× G5 buffer for 3 h at 37°C. The protein extracts were then analyzed by immunoblotting with anti-BRI1 or anti-myc antibodies.

### Yeast growth conditions, transformation and complementation assays

The yeast BY4741 wild type (*MATa; his3*Δ*1 leu2*Δ*0 met15*Δ*0 ura3*Δ*0*) and Δ*yos9* (Y03993; *Mata his3*Δ*1 leu2*Δ*0 met15*Δ*0 ura3*Δ*0* YDR057 *yos9*::kanMX4) strains are from the EUROSCARF collection. The complementation assay was performed using the yeast ERAD substrate CPY* as previously described (Jakob et al. 1998). Briefly, yeast strains were transformed with the *Bgl*II-linearized integrating plasmid pRS306-prc1-1 (Knop et al. 1996) followed by selection on plates containing 5-fluoroorotic acid. For screening of colonies, a fragment of the *PRC1* locus was amplified with primers Sc_CPY_1F/_5R and positive clones were identified by resistance of the PCR fragment towards *Bst*XI digestion. Sequencing of the PCR fragment with primer Sc_CPY_5R confirmed the G255R mutation of *prc1*-*1*. The *yos9*::kanMX4 disruption was confirmed by PCR with primers Sc_Yos9_1F/KanB, Sc_Yos9_2R/KanC, KanD/KanE and Sc_Yos9_1F/_2R.

For the yeast complementation assay, the vector pADHfw containing the ADH1 promoter and a LEU2 marker was used. As a positive control, the coding sequence of the yeast *YOS9* gene was amplified by PCR with primers Sc_Yos9_7F/_8R, *Xho*I/*Bam*HI-digested and ligated into *Xho*I/*Bam*HI-digested pADHfw. The Arabidopsis *OS9* coding sequence was amplified by PCR from cDNA with primers At5g35080_9F/_10R and cloned in the same way. The Arabidopsis *OS9* coding sequence with a C-terminal HDEL motif was created by PCR from the plasmid p20-AtOS9-GFP with the primers At5g35080_22F/_21R, *Xho*I*/Bam*HI-digested and ligated into pADHfw. For generation of the plasmid expressing the full-length *OS9* sequence fused to the C-terminal part of YOS9, the *OS9* open reading frame was amplified with primers At5g35080_22F/-rev-SalI, while the region encoding the C-terminal region of YOS9 was amplified from genomic yeast DNA with primers Sc_Yos9_8R/-fw-SalI. Both PCR fragments were subcloned, cut out by *Bam*HI/*Sal*I- and *Xho*I/*Sal*I-digestion, respectively and ligated together by their compatible *Sal*I sites. The ligation product was used as a template for PCR with primers At5g35080_22F/Sc_Yos9_8R, *Xho*I/*Bam*HI-digested and ligated into pADHfw.

### Analysis of CPY* degradation

To analyze the CPY* degradation, a cycloheximide assay was performed as described (Clerc et al. 2009). Briefly, yeast cells were grown at 30°C to mid-log phase (OD_600_ of 1.0–1.2) and the chase was started by addition of cycloheximide (200 μg mL^−1^; Sigma–Aldrich). 6 × 10^8^ cells were removed at each time point (0, 40, 80 and 120 min), transferred to ice-cold NaN_3_ (10 mM) and harvested by centrifugation. After addition of 150 μL of ice-cold lysis buffer (50 mM Tris–HCl, pH 7.5, 1% SDS, 1 mM EDTA, 1 mM PMSF), the cells were disrupted by vigorous shaking for 2 min at 4°C with 100 μL glass beads. Proteins were subjected to 10% SDS–PAGE, followed by immunoblotting with anti-CPY antibody (Invitrogen). A ChemiDoc Imager (Bio-Rad) was used for signal detection and the Quantity One program (Version 4.6.9) for quantification.

## Results

### Identification of Arabidopsis MRH domain proteins

**Fig. 1:**
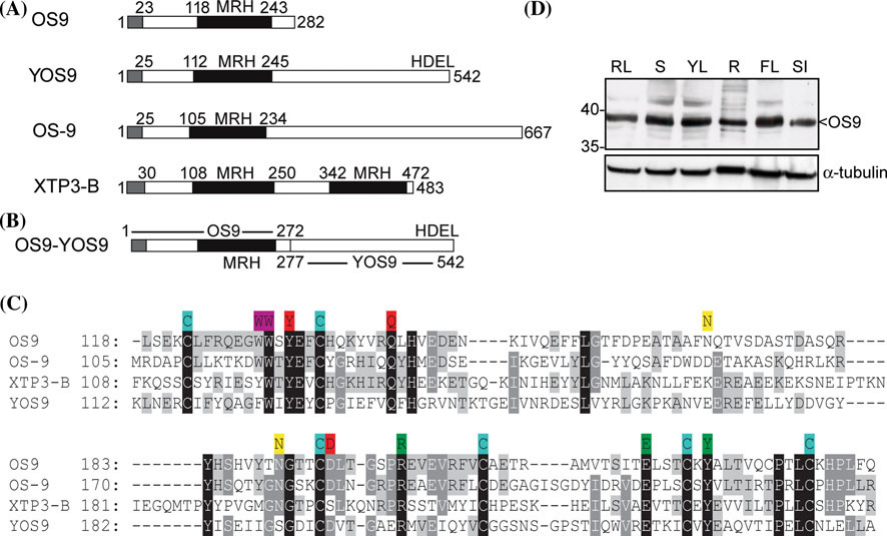
OS9 is a MRH domain protein that is ubiquitously expressed. **a** A comparison of the domain organization of OS9 with yeast (YOS9) and human (OS-9/XTP3-B) MRH domain proteins is shown. The signal peptide is indicated in *grey*, the MRH domain is shown in *black*. For OS-9 and XTP3-B the longest variants are shown (Hosokawa et al. 2010). **b** The chimeric OS9-YOS9 protein that was used for complementation of Δ*yos9* yeast consists of amino acids 1–272 from Arabidopsis OS9 fused to amino acids 277–542 from yeast YOS9. **c** Sequence alignment of MRH domains was performed using ClustalW (http://www.ebi.ac.uk/Tools/clustalw2/index.html). The domains were identified according to Munro (2001). Conserved residues are *shaded* (100% identity *black*, 80% identity *dark grey*, 60% identity *light grey*). The six conserved cysteines involved in the formation of disulphide bonds are marked in *blue*, amino acid residues involved in carbohydrate binding are marked in *red* (Roberts et al. 1998; Szathmary et al. 2005) and putative N-glycosylation sites are marked in *yellow*. The two tryptophan residues that determine the α1,6-mannose binding specificity of human OS-9 are marked in magenta (Satoh et al. 2010) and the three mannose binding residues of YOS9 are marked in *green* (Ghosh et al. 2003; Bhamidipati et al. 2005). **d** Proteins were extracted from different *A. thaliana* organs or developmental stages (*RL* rosette leaves, *S* whole seedlings, *YL* young leaves, *R* root, *FL* flower, *SI* siliques), separated by SDS–PAGE and analyzed by immunoblots using OS9-specific antibodies. α-tubulin expression was used as a control

The Arabidopsis genome contains two MRH domain-containing proteins. One is annotated as the α-glucosidase II β-subunit, which is involved in removal of terminal α1,3-glucose residues during N-glycan trimming and the calnexin/calreticulin cycle (Quinn et al. 2009; Soussilane et al. 2009; D‘Alessio et al. 2010). The second Arabidopsis MRH domain protein (At5g35080) herein referred to as OS9 is a 282 amino acid long protein with a predicted signal peptide and three putative N-glycosylation sites (N94, N169, N190). The amino acid sequence of the OS9 MRH-region has 27% identity to YOS9 and 37 and 31% identity to human OS-9 and XTP3-B, respectively. In contrast to YOS9 the Arabidopsis OS9 protein does not contain a C-terminal HDEL motif (Fig. 1a) and the C-terminal domain that is present in YOS9 and human OS-9 (Hosokawa et al. 2010; Satoh et al. 2010) is completely missing in OS9. In contrast, the six cysteine residues involved in disulfide bridge formation and the residues of the carbohydrate binding site (Munro 2001; Satoh et al. 2010) are all conserved (Fig. 1c) suggesting that OS9 could be a mannose binding protein with a related function.

To examine the expression pattern of OS9 in *A. thaliana*, protein extracts from different plant organs were subjected to SDS–PAGE and immunoblotting with an OS9-specific antibody. The intensity of the protein band was similar in all analyzed plant organs (Fig. 1d), which is consistent with DNA microarray data (https://www.genevestigator.com/), suggesting that OS9 is ubiquitously expressed during development. Co-expression analysis using ATTED-II (http://atted.jp/) indicated that OS9 is expressed together with ER-located chaperones like SHD, SDF2 and ERdj3B, which are involved in ER quality control processes in plants (Ishiguro et al. 2002; Yamamoto et al. 2008; Nekrasov et al. 2009; Schott et al. 2010).

### OS9 is an ER-located glycoprotein

**Fig. 2:**
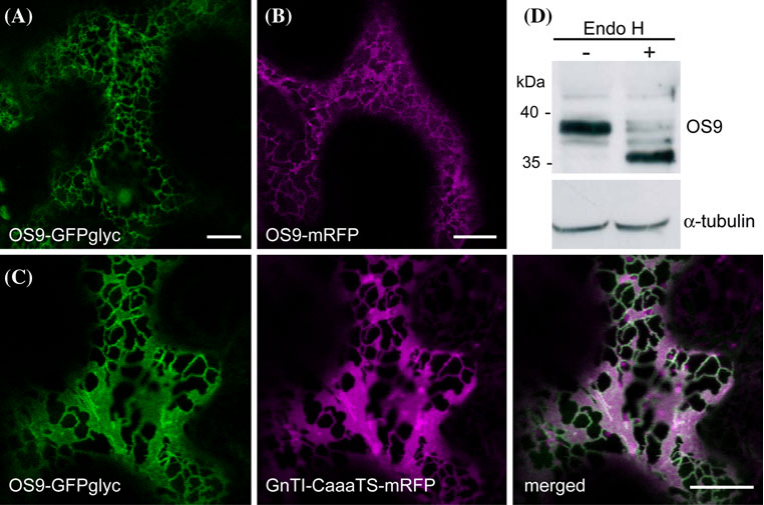
Fluorescent protein-tagged OS9 displays ER localization. Confocal microscopy of *N. benthamiana* leaf epidermal cells expressing **a** OS9-GFPglyc, **b** OS9-mRFP and **c** OS9-GFPglyc (in *green*) together with the ER-retained construct GnTI-CaaaTS-mRFP (in *magenta*) reveals ER localization of OS9 (merged image). *Scale bars* = 10 μM. **d** Immunoblot analysis of the glycosylation status of endogenous OS9. Samples were subjected to Endo H digestion, separated by SDS–PAGE and analyzed by immunoblotting with OS9-specific antibodies or α-tubulin as a control

The absence of any transmembrane domain and known ER-retention/retrieval signals suggest that OS9 is secreted in plants. However, involvement in ERAD requires localization of OS9 in the ER. To determine its subcellular location we fused the fluorescent proteins GFPglyc (Schoberer et al. 2009) and mRFP, respectively, to the C-terminal end of OS9. The resulting OS9-mRFP and OS9-GFPglyc proteins were transiently expressed in *N. benthamiana* leaf epidermal cells and analyzed by confocal laser scanning microscopy. For both constructs a reticulate fluorescent pattern was observed indicating accumulation of the protein in the ER (Fig. 2a, b). Co-expression of OS9-GFPglyc and GnTI-CaaaTS-mRFP, a marker which predominantly labels the ER (Schoberer et al. 2009), showed an overlap of both fluorescent proteins (Fig. 2c). We purified OS9-GFPglyc from *N. benthamiana* leaves by affinity chromatography and analyzed peptides for the presence of N-glycans by LC–ESI–MS. All three N-glycosylation sites of OS9 were occupied with oligomannosidic N-glycans (Fig. S1). Consistently, endogenous OS9 from *A. thaliana* seedlings was sensitive to Endo H digestion. Together these data demonstrate that OS9 is located in the ER (Fig. 2d).

### A chimeric OS9-YOS9 fusion protein suppresses the yeast Δ*yos9* defect

**Fig. 3:**
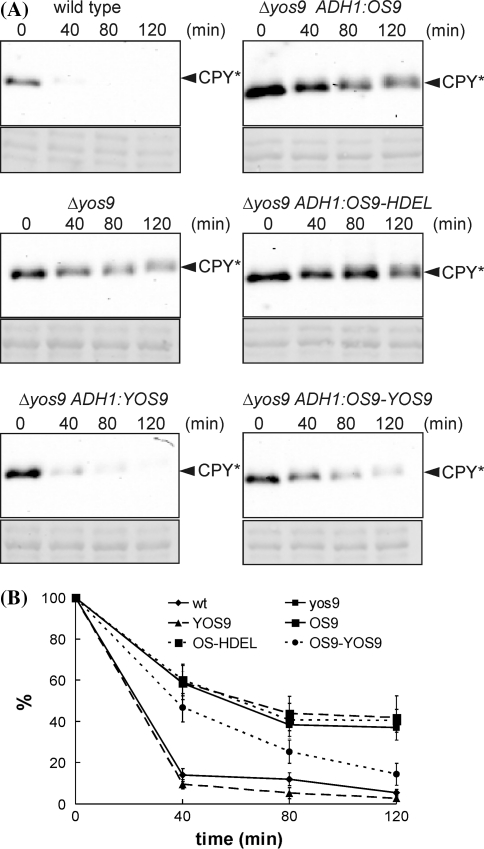
A chimeric OS9-YOS9 protein suppresses the yeast Δ*yos9* defect. **a** Equal cell numbers of yeast strains expressing the ERAD substrate CPY* and OS9 or YOS9 were incubated with cycloheximide and proteins were extracted at the given time points. Shown are representative immunoblot images. The lower panel shows a Ponceau S staining of the membrane. **b** Signal intensities (vertical axis) of the CPY* specific band from three independent experiments were quantified and blotted against time. The amount of the CPY* protein band at time point 0 was set to 100%

A mutated form of the soluble carboxypeptidase Y (CPY*) protein serves as an ERAD substrate in yeast. In wild-type yeast CPY* is rapidly degraded, while deletion of YOS9 in the Δ*yos9* mutant reduces CPY* degradation (Szathmary et al. 2005). To test if Arabidopsis OS9 is the functional homolog of YOS9 we expressed it under a constitutive promoter in Δ*yos9* yeast cells expressing CPY* (Δ*yos9* CPY*). Although the Arabidopsis OS9 protein was expressed in yeast as determined by immunoblotting (data not shown) it did not result in significant degradation of CPY* (Fig. 3a, b). In contrast, the expression of YOS9, which served as a control, led to profound degradation of CPY* during the cycloheximide incubation period. This finding suggests that Arabidopsis OS9 cannot substitute for YOS9. Possible explanations could be that OS9 is not properly retained in the ER of *S. cerevisiae* since it does not contain a HDEL/KDEL retrieval signal, or that the C-terminal part of YOS9 is required for its function in yeast. To test these two possibilities we generated one HDEL-tagged OS9 form (OS9-HDEL) and a chimeric form composed of the C-terminal domain of YOS9 (amino acids 277–542) fused to OS9 (OS9-YOS9) (Fig. 1b). Whilst expression of OS9-HDEL did not result in an improved degradation of CPY* (Fig. 3a, b), OS9-YOS9 increased the degradation of CPY*. In summary, these results show that Arabidopsis OS9 cannot complement the CPY* degradation defect of the yeast Δ*yos9* mutant, because it lacks the C-terminal domain of YOS9, that is required for association with the membrane-embedded HRD3-HRD1 ubiquitin ligase complex (Gauss et al. 2006).

### The UPR is activated in the *os9-1* mutant

**Fig. 4:**
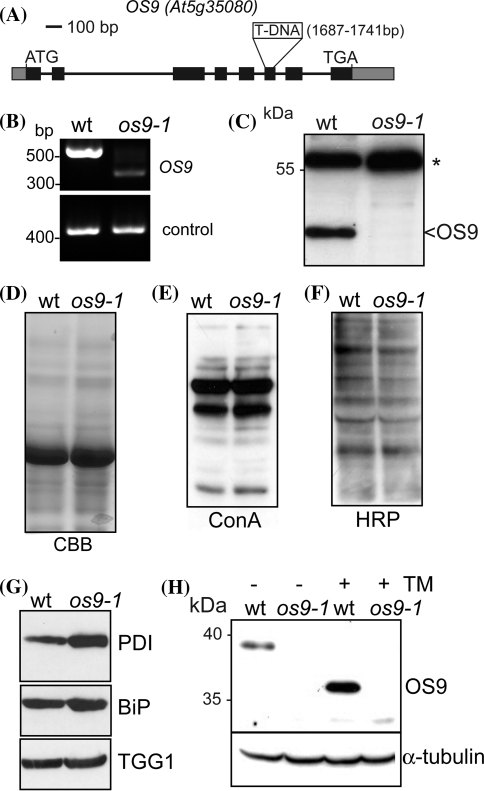
*os9*-*1* lacks a functional OS9 protein and displays activation of the UPR. **a** Schematic overview of the OS9 gene structure. *Boxes* represent exons (the *black area* represents the coding region), the *os9*-*1* T-DNA insertion is indicated. **b** Reverse transcription-PCR analysis of the *os9*-*1* mutant using oligonucleotides that flank the insertion site. UBQ5 amplification served as a control. **c** Immunoblot with anti-OS9 antibodies. The *asterisk* indicates an unspecific band, which was used as a loading control. **d** Protein extracts from wt and *os9*-*1* 14-day-old seedlings were subjected to SDS–PAGE and Coomassie brilliant blue (CBB) staining. **e** Blots were analyzed using concanavalin A (ConA) (**f**) or anti-horseradish peroxidase (HRP) antibodies, which recognize N-glycans with β1,2-xylose and core α1,3-fucose residues. **g** Protein extracts were analyzed by immunoblotting using anti-PDI, anti-BiP2 and anti-TGG1 antibodies, respectively. **h** 10-day-old seedlings were incubated for 24 h in ½× MS medium supplemented with 3% sucrose and 5 μg mL^−1^ tunicamycin (TM), protein extracts were analyzed using OS9 or α-tubulin antibodies

We searched in the mutant collections for putative OS9 knockout lines and identified the SALK_029413 line. We screened the segregating population and identified a homozygous T-DNA insertion line termed *os9*-*1*. One of the T-DNA insertion sites in *os9*-*1* was located at an intron–exon junction (Fig. 4a) resulting in the formation of a smaller transcript derived from an incorrect splicing event (Fig. 4b). The aberrant transcript completely lacks exons 6 and 7 of the *OS9* gene but contains a functional open reading frame leading to a protein with a 58 amino acid deletion. In addition we identified low amounts of another aberrant transcript in *os9*-*1* which harbours a deletion of exon 6 resulting in the formation of a transcript with a premature stop codon. By immunoblotting a band corresponding to the full-length OS9 protein or truncated versions thereof could not be detected in the *os9*-*1* mutant. Moreover, since the deletion of exons 6 and 7 affects almost half of the MRH domain, the *os9*-*1* line very likely does not produce a functional OS9 protein and thus represents a null allele (Fig. 4c).

To monitor whether OS9 deficiency results in altered N-glycosylation or differences in protein levels we extracted proteins from *os9*-*1* leaves and subjected them to SDS-PAGE and immunoblotting. No proteins with altered migration were detected by Coomassie staining of protein extracts (Fig. 4d). The analysis using antibodies against complex N-glycans and lectin overlays with concanavalin A (ConA) did not reveal any drastic changes in the staining pattern (Fig. 4e, f). Total N-glycan analysis of protein extracts from leaves showed that the overall composition of N-glycans was similar to wild type in *os9*-*1*, with a minor increase of Man_5_GlcNAc_2_ structures and a decrease of truncated processed (MMXF) N-glycans (Fig. S2). These findings strongly indicate that OS9 is not directly involved in N-glycan processing.

Underglycosylation of particular proteins leads to a change in mobility when analyzed by SDS–PAGE. However, neither the ER-resident protein disulfide isomerase (PDI) nor the thioglucoside glucohydrolase TGG1, which have both been shown to be affected in mutants with an underglycosylation defect (Koiwa et al. 2003; Lerouxel et al. 2005; Farid et al. 2011), displayed altered mobility in *os9*-*1* (Fig. 4g). However, PDI and binding protein 2 (BiP2) protein levels were increased in *os9*-*1*, indicating the activation of the UPR due to the absence of a functional OS9 protein.

We also tested whether the UPR increases OS9 protein levels. Seedlings were incubated with tunicamycin and OS9 expression was monitored by immunoblotting. A clear shift in mobility as well as upregulation of OS9 was observed in wild-type plants treated with tunicamycin (Fig. 4h) indicating that OS9 expression is induced by the UPR.

### OS9 deficiency suppresses the dwarf phenotype of *bri1-5* and *bri1-9* mutants

**Fig. 5:**
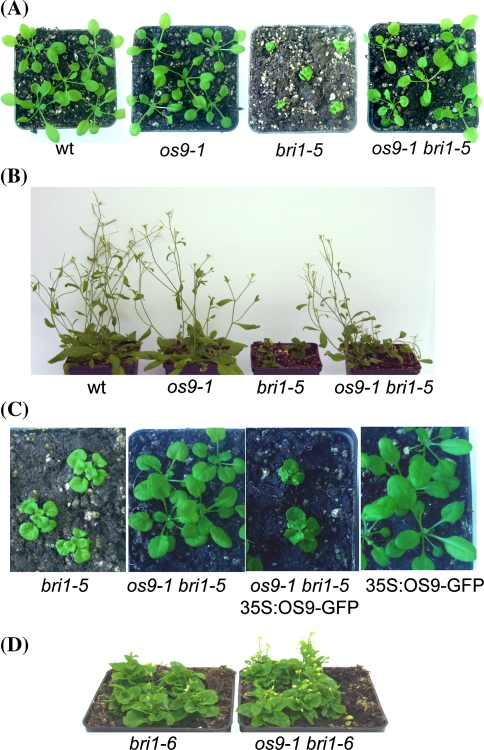
*os9*-*1* suppresses the *bri1*-*5* phenotype by affecting the ER-retention of BRI1-5. **a** 17-day and **b** 4-week-old soil-grown plants. **c** Expression of OS9-GFP in *os9*-*1 bri1*-*5* plants restores the *bri1*-*5* growth phenotype. *os9*-*1 bri1*-*5* double mutants were floral dipped with 35S:OS9-GFP. Expression of 35S:OS9-GFP in wild-type is shown as a control. **d** 10-week-old *bri1*-*6* single and *os9*-*1 bri1*-*6* double mutants display the *bri1*-*6* dwarf phenotype

It has been reported that the dwarf phenotype of *bri1*-*5* can be rescued by kifunensine treatment or by mutants that block the ERAD pathway (Hong et al. 2009; Liu et al. 2011; Su et al. 2011). We suspected that OS9 could play a crucial role in ERAD of glycoproteins in plants similar to YOS9 and human OS-9/XTP3-B. To test this hypothesis we crossed *os9*-*1* to *bri1*-*5* and analyzed the double mutant for phenotypic changes. *os9*-*1* was able to partially suppress the *bri1*-*5* phenotype when seedlings were grown under light/dark growth conditions (Fig. S3) and almost completely restored the growth defect of plants cultivated on soil (Fig. 5a, b). Ectopic expression of OS9-GFP in *os9*-*1 bri1*-*5* resulted in the generation of transgenic plants with the dwarf phenotype (Fig. 5c and Fig. S4) strongly indicating that a functional OS9 protein interferes with the maturation of BRI1-5.

Apart from BRI1-5 it was also shown that BRI1-9, another mutated BRI1 form, is retained in the ER and only released to the plasma membrane upon blockage of the ERAD pathway or disturbance of the ER quality control system (Jin et al. 2007; Hong et al. 2008, 2009). Consistent with our prediction, *os9*-*1* partially suppresses the dwarf phenotype of *bri1*-*9* (Fig. S5).

To show that the suppression of the phenotype is allele-specific we crossed *os9*-*1* to *bri1*-*6*, which is another *bri1* allele with a missense mutation (G644D) in the brassinosteroid binding domain (Noguchi et al. 1999) that does not result in ER retention of the mutated BRI1-6 protein, but very likely interferes with intra-molecular signal transduction of the brassinosteroid receptor (Kinoshita et al. 2005; Hong et al. 2008). As shown in Fig. 5d and Fig. S6 *os9*-*1* fails to rescue the *bri1*-*6* defect suggesting that OS9-mediated rescue of the *bri1* growth defect is specific for the ER-retained forms of the mutated BRI1 receptor.

### Interaction of BRI1-5 and BRI1-9 protein with OS9

**Fig. 6:**
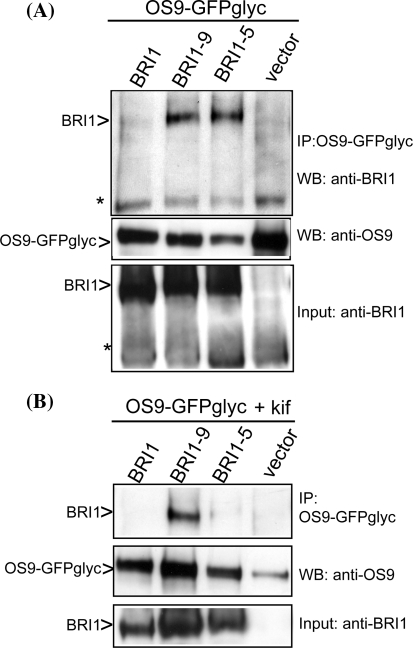
OS9 interacts with the ERAD substrates BRI1-5 and BRI1-9. **a** BRI1 forms were transiently co-expressed with OS9-GFPglyc in *N. benthamiana* leaves. OS9-GFPglyc was purified and co-purified protein fractions were analyzed by SDS–PAGE and immunoblotting with anti-BRI1 and anti-OS9 antibodies. The asterisk indicates an unspecific band that was used as loading control. **b** As in **a** but expression was done in the presence of 20 μM kifunensine (kif)

Our data indicate that OS9 is involved in degradation of ER-retained BRI1 proteins. To investigate whether OS9 interacts with BRI1 proteins we expressed different BRI1 forms together with OS9-GFPglyc transiently in *N. benthamiana* leaves, purified the OS9-GFPglyc by Protein A affinity chromatography and performed immunoblots with anti-BRI1 antibodies to analyze the binding to OS9. In contrast to wild-type BRI1, BRI1-5 and BRI1-9 could be co-purified with OS9-GFPglyc (Fig. 6a). Like OS9, BRI1-5-GFP was found in the ER and displayed Endo H sensitive N-glycans, indicating that the interaction of OS9 and BRI1-5 takes place in the ER (Fig. S7).

In the presence of kifunensine association of OS9 with BRI1-5 was clearly reduced, being consistent with the idea that OS9 binding to ERAD substrates requires recognition of a distinct mannose residue generated by α-mannosidases (Fig. 6b). Interestingly the interaction of BRI1-9 was more resistant to kifunensine treatment highlighting differences between the two ERAD substrates that have also been described previously (Jin et al. 2007; Hong et al. 2008, 2009; Jin et al. 2009). To further investigate the role of the putative MRH domain for its function and interaction with ERAD clients we generated a mutant OS9 version where the arginine residue at position 201, which corresponds to R200 in YOS9, was mutated to alanine (OS9R201A mutant). For YOS9 it has been shown that this residue is critical for its lectin binding ability and subsequent ERAD of misfolded proteins (Bhamidipati et al. 2005; Szathmary et al. 2005; Quan et al. 2008). Consistent with our prediction the expression of OS9R201A-GFPglyc in *os9*-*1 bri1*-*5* did not restore the dwarf phenotype of *bri1*-*5* (Fig. S8).

### OS9 expression is drastically reduced in the *sel1l* mutant

**Fig. 7:**
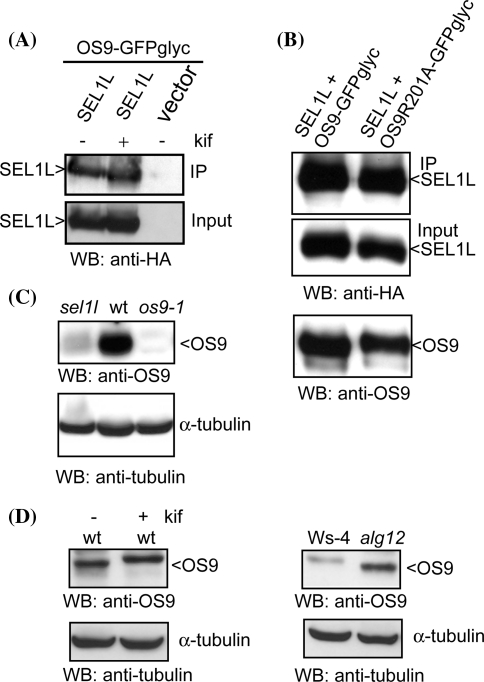
OS9 interacts with SEL1L in a glycan-independent way. **a** HA-tagged *A. thaliana* SEL1L was co-expressed with OS9-GFPglyc in *N. benthamiana*. OS9-GFPglyc was purified and co-purified protein fractions were analyzed by immunoblotting with anti-HA antibodies. (+) and (−) indicates expression in the presence or absence of 20 μM kifunensine (kif). **b** As in **a** but the mutated OS9R201A-GFPglyc was used for purification of SEL1L. **c** Protein extracts from *A. thaliana sel1l*, wild-type (wt) and *os9*-*1* were separated by SDS–PAGE and analyzed by immunoblotting with anti-OS9 antibodies. α-tubulin expression was used as a control. **d** As in **c** but wt with kif and *alg12* are shown. *alg12* is in Ws-4 background. OS9 is more abundant in *alg12* than in Ws-4, which is very likely caused by the activation of the unfolded protein response in *alg12* (Hong et al. 2009)

For mammalian cells it has been shown that SEL1L and OS-9 interact and it has been suggested that this interaction is dependent on the N-glycans present on SEL1L (Gauss et al. 2006; Christianson et al. 2008). Consequently, we asked the question whether *A. thaliana* SEL1L and OS9 can also associate. To this end we co-expressed OS9-GFPglyc and SEL1L containing a 3× HA-tag transiently in *N. benthamiana* and performed co-purification experiments. We observed that OS9 and SEL1L displayed a strong association. Importantly, this interaction was independent of kifunensine treatment (Fig. 7a) and was also found when the mutated OS9R201A-GFPglyc was used to co-purify SEL1L-HA (Fig. 7b).

To see whether OS9 protein levels are altered in the absence of SEL1L (Liu et al. 2011; Su et al. 2011) we performed immunoblots with protein extracts from *A. thaliana* *sel1l* knockout seedlings (Fig. 7c). OS9 levels were considerably reduced in seedlings suggesting that SEL1L is required for OS9 stability or ER retention due to association in a complex. We investigated whether the interaction between OS9 and SEL1L is dependent on a defined glycan structure present on SEL1L, which is glycosylated in *A. thaliana* (Su et al. 2011). If so, the interaction should be abolished by kifunensine treatment or in glycosylation mutants like *alg12* which produce aberrant N-glycan structures that block the ERAD pathway (Hong et al. 2009). OS9 protein levels were unchanged in wild-type seedlings in the presence of kifunensine as well as in *alg12* plants (Fig. 7d). Together these data indicate that mannose trimming and the exposure of α1,6-mannosyl residues is not required for SEL1L-OS9 interaction.

### The os9-1 mutant displays a salt stress phenotype

**Fig. 8:**
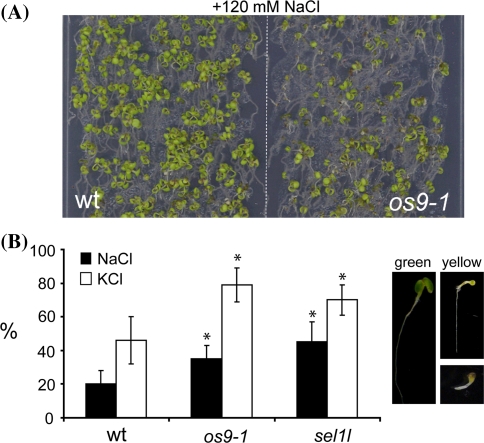
*os9*-*1* seedlings are sensitive to salt stress. **a** Wild-type (wt) and *os9*-*1* seedlings were directly spread on ½× MS medium containing 1.5% sucrose supplemented with 120 mM NaCl and grown for 12 days. **b** Quantitative analysis of different seedling phenotypes grown for 12 days either on 120 mM NaCl or KCl. Percentages (vertical axis) represent smaller and yellow seedlings and are means ± SE from three independent repeats (more than 100 seedlings were counted per line and experiment, the total number of seedlings represents 100%). *P < 0.05 (paired Student’s *t* test)

Recently it was shown that ERAD is necessary for plant salt tolerance as *sel1l* knockout seedlings displayed increased salt sensitivity (Liu et al. 2011). We subjected *os9*-*1* plants to different salt treatments and analyzed the phenotype. Germination of seeds on MS medium with 120 mM NaCl showed that *os9*-*1* seedlings are more sensitive towards NaCl than wild type (Fig. 8a). Moreover, *os9*-*1* and *sel1l* seedlings were also more sensitive towards KCl (Fig. 8b) corroborating the finding that disruption of the ERAD pathway results in increased salt sensitivity. We also tested whether OS9 is important for the function of the heavily glycosylated receptor kinase EFR, which is required for the perception of the bacterial EF-Tu during pathogen infection (Zipfel et al. 2006). In previous studies it was shown that EFR is a client of the *A. thaliana* ER quality control system and mutations in diverse ER-chaperones and glycosylation enzymes affect its function (Li et al. 2009; Nekrasov et al. 2009; Saijo et al. 2009; Häweker et al. 2010). To test the effect of OS9 deficiency we performed a seedling growth inhibition assay (Fig. S9) in the presence of the bacterial peptide elf18. The *os9*-*1* seedlings displayed a retarded growth phenotype similar to wild type indicating that OS9 is not required for EFR function.

It has also been shown that *sel1l* knockout plants are more sensitive to tunicamycin, which leads to underglycosylation of proteins and subsequent activation of the UPR, and to paraquat, an inducer of oxidative stress (Liu et al. 2011). However, *os9*-*1* seedlings were less sensitive than *sel1l* mutants to both chemicals and appeared indistinguishable from wild-type seedlings (Fig. S10).

## Discussion

The ERAD machinery identifies and degrades individual aberrant proteins and ensures together with the protein folding machinery that only properly folded and assembled proteins are released from the ER. The disposal of misfolded proteins is crucial to prevent the accumulation of defective proteins in the ER, which compromise its function leading to ER stress and eventually cell death. Deletion of ERAD components results in systemic ER stress and embryo lethality in mammals (Francisco et al. 2010). Despite its importance for protein homeostasis in the cell very little is known about the ERAD pathway in plants (Vitale and Boston 2008; Liu and Howell 2010a; Ceriotti 2011). The recent identification of the *A. thaliana* SEL1L/HRD3 and HRD1 homologs has highlighted the importance of ERAD components for cellular processes (Liu et al. 2011; Su et al. 2011). Here, we provide evidence that the so far uncharacterized *A. thaliana* OS9 protein functions in the glycoprotein ERAD pathway: (i) *A. thaliana* OS9 contains an MRH domain with homology to mammalian and yeast proteins involved in ERAD of glycosylated substrates. (ii) OS9 is an ER-located protein. (iii) OS9 containing the C-terminal domain of YOS9 can complement the Δ*yos9* yeast mutant. (iv) OS9 can suppress the *bri1*-*5* and *bri1*-*9* growth phenotypes and (v) interacts with these heavily glycosylated ERAD substrates as well as with the *A. thaliana* SEL1L protein. The association of OS9 with BRI1-5 is dependent on trimming of mannose residues and presumably requires an intact MRH domain. SEL1L, on the other hand, is involved in the degradation of glycosylated as well as non-glycosylated proteins presumably by association with misfolded protein domains exposed on ERAD substrates (Liu et al. 2011; Su et al. 2011).

The effect of OS9 deficiency on the stability of ERAD substrates remains to be shown. The suppression of the *bri1-5* and *bri1-9* dwarf phenotypes by OS9 deficiency indicates that BRI1-5 and BRI1-9 proteins can escape from the ER either due to accumulation in the absence of a specific degradation process or due to lack of OS9-specific ER-retention. We have not yet identified the oligosaccharide structure that constitutes, together with the misfolded polypeptide domain, the recognition signal for OS9. The presence of conserved residues in the MRH domain including the double tryptophan motif that has been suggested to confer specificity of human OS-9 for the terminal α1,6-mannose (Satoh et al. 2010) and the finding that ERAD is blocked in the Arabidopsis *alg12* mutant that lacks this sugar residue (Hong et al. 2009) suggest that OS9 has a similar oligosaccharide binding specificity as found for yeast and mammalian homologs (Quan et al. 2008; Hosokawa et al. 2009). However, our data also indicate that there are fundamental variations between the different systems. OS9 can only restore ERAD of CPY* in Δ*yos9* yeast cells when it contains the C-terminal YOS9 domain, and the expression of YOS9 in Arabidopsis *bri1*-*5 os9*-*1* mutants does not revert the *bri1*-*5 os9*-*1* phenotype (data not shown). For YOS9 it has been shown that a region within amino acids 250–420 in its C-terminal domain is required for interaction with HRD3 (Gauss et al. 2006) and together YOS9 and HRD3 ensure that only terminally misfolded proteins are degraded. OS9 lacks this region, which can explain its non-function in yeast.

The molecular mechanisms of the glycan-dependent steps in the mammalian ERAD pathway are still unclear and two models have been proposed for the role of N-glycans and the ER-resident OS-9/XTP3-B lectins (Hebert et al. 2010; Hosokawa et al. 2010). In the more widely favoured model an oligosaccharide on a misfolded protein acts like in yeast as the degradation signal and is recognized by these lectins. The second model proposes that the OS-9/XTP3-B-SEL1L interaction is mediated by binding of the MRH domain to the N-glycans of SEL1L and association with ERAD substrates is independent of oligosaccharide binding (Christianson et al. 2008). In agreement with the former model, our data indicate that SEL1L-OS9 interaction in plants is glycan-independent, because interaction persisted when mannose trimming was abolished and the MRH domain was mutated. Since OS9 lacks any C-terminal YOS9 domain that is required for association with HRD3 our finding raises the so far unsolved question how OS9 associates with Arabidopsis SEL1L/HRD3 and the HRD-ligase complex that links substrate recognition with proteasomal degradation. Moreover, since OS9 protein levels are clearly reduced in the *sel1l* mutant we propose that OS9-SEL1L interaction plays also a role for the ER retention of OS9, which lacks any obvious ER-retention or retrieval signal.

Here, we show that OS9 interacts with both BRI1-5 and BRI1-9, but kifunensine treatment disturbed the association with BRI1-5 much more than with BRI1-9. The two distinct mutations affect very different regions of BRI1 (Hothorn et al. 2011; She et al. 2011) and genetic as well as biochemical studies have shown that the two mutated BRI1 forms have different requirements for ER quality control (Jin et al. 2007; Hong et al. 2008). Consequently our data argue that BRI1-5 and BRI1-9 represent different classes of ERAD substrates that require distinct components for degradation like it has also been proposed for mammalian ERAD substrates (Bernasconi et al. 2010). The interaction of BRI1-9 with OS9 might be more dependent on interaction with exposed protein segments and OS9 may—like YOS9—also bind and assist in degradation of certain non-glycosylated substrates (Bhamidipati et al. 2005; Jaenicke et al. 2011).

Which endogenous proteins are normally recognized by OS9 and selected for degradation through the ERAD pathway? The *os9*-*1* mutant does not display any morphological phenotype and the overall protein levels do not appear drastically changed in the mutant. However, under abiotic stress conditions the *os9*-*1* mutant displays a clear phenotype, which is in agreement with data for *sel1l* and the finding that salt stress leads to ER stress and presumably accumulation of misfolded proteins that have to be removed in order to prevent damage to the cells (Liu and Howell 2010a). Moreover, UPR triggered by tunicamycin or DTT treatment induces OS9 expression (Martínez and Chrispeels 2003; Liu and Howell 2010b), but interestingly, *os9*-*1* seedlings display a similar response like wild type when treated with tunicamycin. Clearly, additional studies are needed to identify endogenous plant ERAD substrates and to dissect the important role of ERAD components under different stress conditions. We are still at the beginning of understanding this process for plants and our knowledge is restricted by the limited number of available plant ERAD substrates. However, the characterization of OS9 together with the recently identified ERAD components will help to elucidate the mechanism as well as the biological function of the pathway in the future.

## Electronic supplementary material

Below is the link to the electronic supplementary material.

Supplementary material 1 (PDF 13,430 kb)
