# Supplementary material for: Unraveling the function of Arabidopsis thaliana OS9 in the endoplasmic reticulum-associated degradation of glycoproteins
Source: Plant Mol Biol. 2012 Feb 11;79(1):21–33. doi: 10.1007/s11103-012-9891-4 (PMC3332353; doi:10.1007/s11103-012-9891-4)
Supplement: Supplementary file 1 — Supplementary material 1 (PDF 13,430 kb) [file 11103_2012_9891_MOESM1_ESM.pdf]

Electronic supplementary material for:

Unraveling the Function of *Arabidopsis thaliana* OS9 in the Endoplasmic Reticulum-Associated Degradation of Glycoproteins

Plant Molecular Biology

Silvia Hüttner, Christiane Veit, Jennifer Schoberer, Josephine Grass and Richard Strasser

Corresponding Author: Richard Strasser
e-mail: richard.strasser@boku.ac.at
phone: +43 1 47654 6705
fax: +43 1 47654 6392

Table S1. Oligonucleotide sequences as used in this study.
Fig. S1. LC-ESI-MS of OS9-GFPglyc purified from *N. benthamiana* leaves.
Fig. S2. Matrix-assisted laser desorption ionization time-of-flight mass spectrometry of N-glycans extracted from *A. thaliana* leaves.
Fig. S3. *os9-1* partially suppresses the *bri1-5* phenotype.
Fig. S4. Expression of OS9-GFP and OS9-GFPglyc in *os9-1 bri1-5* plants restores the *bri1-5* phenotype.
Fig. S5. *os9-1* partially suppresses the *bri1-9* phenotype.
Fig. S6. *os9-1* does not suppress the *bri1-6* phenotype.
Fig. S7. BRI1-5-GFP is retained in the ER.
Fig. S8. Expression of OS9R201A-GFPglyc does not restore the *bri1-5* phenotype in *os9-1 bri1-5* plants.
Fig. S9. *os9-1* seedlings are sensitive to elf18.
Fig. S10. Effect of tunicamycin and paraquat on *os9-1* seedlings.

Table S1. Oligonucleotide sequences as used in this study

Name	Sequence (5’ – 3’)
LBa1	TGGTTCACGTAGTGGGCCATCG
At5g35080_1F	TCTAATGGTATGGCCCTTGTATCCTG
At5g35080_2R	GTTCTTCCTTGTTTCTTGTTGCGTCTT
At5g35080_3F	AAAAAGCGACGAGTGGATGGAC
At5g35080_4F	TTCTAGAATGAGAATCACGCAGATCTTGTT
At5g35080_5R	TGGATCCAGAATCAGCTATCATCTTAGGTG
At5g35080_6F	CCGTCCGGTGCTATTCTCCTTC
At5g35080_7R	AGCGGGTAACACTGAAAATGGAGATA
At5g35080_21R	tattCTCGAGTCAGAGTTCGTCGTGAGAATCAGCTATCATCTTAGGTG
At5g35080_22F	tataGGATCCATGAGAATCACGCAGATCTTGTT
At5g35080-rev-SalI	TATAGTCGACTGCTTGTTCTTCCTTGTTTCT
At5g35080_23F	ATCTTACAGGATCACCTGCCGAAGTCGAGGTGAGGT
At5g35080_23R	ACCTCACCTCGACTTCGGCAGGTGATCCTGTAAGAT
At1g18260_1F	AGTAGCAGAAAGGGGGCCGTGGAGT
At1g18260_2R	GAGAATCAGGCGACAAGTATCAAGTTT
At1g18260_10F	TACTAGTATGAGAATATTAAGCTACGGAATCG
At1g18260_11R	TAGATCTCCGTGGGAACGCAGCGAGGTGTT
BRI1_1F	tGGATCCATGAAGACTTTTTCAAGCTTCTTTCTCT
BRI1_2R	tGGATCCTAATTTTCCTTCAGGAACTTCTTTTATAC
BRI1_7F	GTTTCTGGACATGTTTTACAACATGTTGT
BRI1_7R	ACAACATGTTGTAAAACATGTCCAGAAAC
BRI1_10F	TACGTTGTTGAAGATGAGAGGACT
BRI1_13F	CAAAAACGATGGGATGAAGAAAGAGT
BRI1_14R	ACACCGCGGAAGAGGATAACCACAG
BRI1_15F	TTCTTCTTCTCCTTCTTTTCTCTTTCATT
BRI1_16R	ACGACGTTAGCACCGGAGATTGAA
BRI1_17F	tataACTAGTATGAAGACTTTTTCAAGCTTCTTTCTC
BRI1_18R	tataGGATCCGATTTTGTTTCCGCTAATCGCTAA
Sc_CPY_1F	CCATCGGGAACCCTTACTCTTG
Sc_CPY_5R	CACAGGCCATTGGTTCGTAA
Sc_Yos9_1F	ATTCCTATATTTGGTTGGGTTTTTAAG
Sc_Yos9_2R	ATGAACAAATTCTATGCCAGGACAGTA
Sc_Yos9_7F	AGGATCCATGCAAGCTAAAATTATATATGCTCTG
Sc_Yos9_8R	ACTCGAGTTAAAGCTCATCATGTTCGATGAA
Sc_Yos9-fw-SalI	TAATGCCGTCGACTATGAACCGATTTTTTTAG
KanB	CTGCAGCGAGGAGCCGTAAT
KanC	TGATTTTGATGACGAGCGTAAT
KanD	CCCCGGCAAAACAGCATTCC
KanE	TCACCGAGGCAGTTCCATAGG

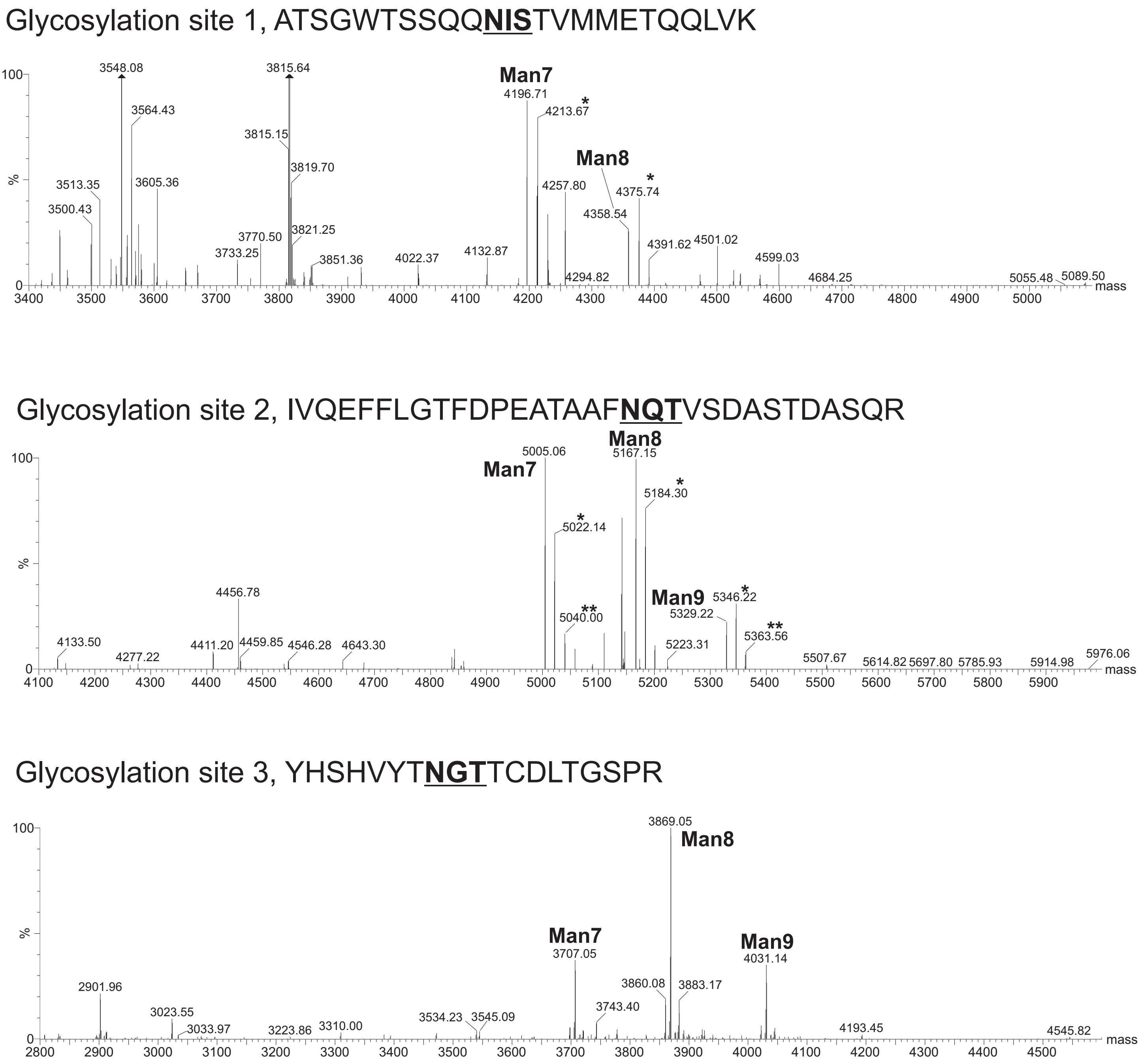

Fig. S1. LC-ESI-MS of OS9-GFPglyc purified from *N. benthamiana* leaves. Mass spectra of the three glycopeptides derived from *A. thaliana* OS9 are shown. Man7, Man8 and Man9 are oligomannosidic peaks corresponding to Man7GlcNAc2, Man8GlcNAc2 and Man9GlcNAc2. The asterisks indicate ammonia adducts of the same peaks. The whole procedure for purification and detection of glycopeptides has been described previously (Schoberer et al., 2009).

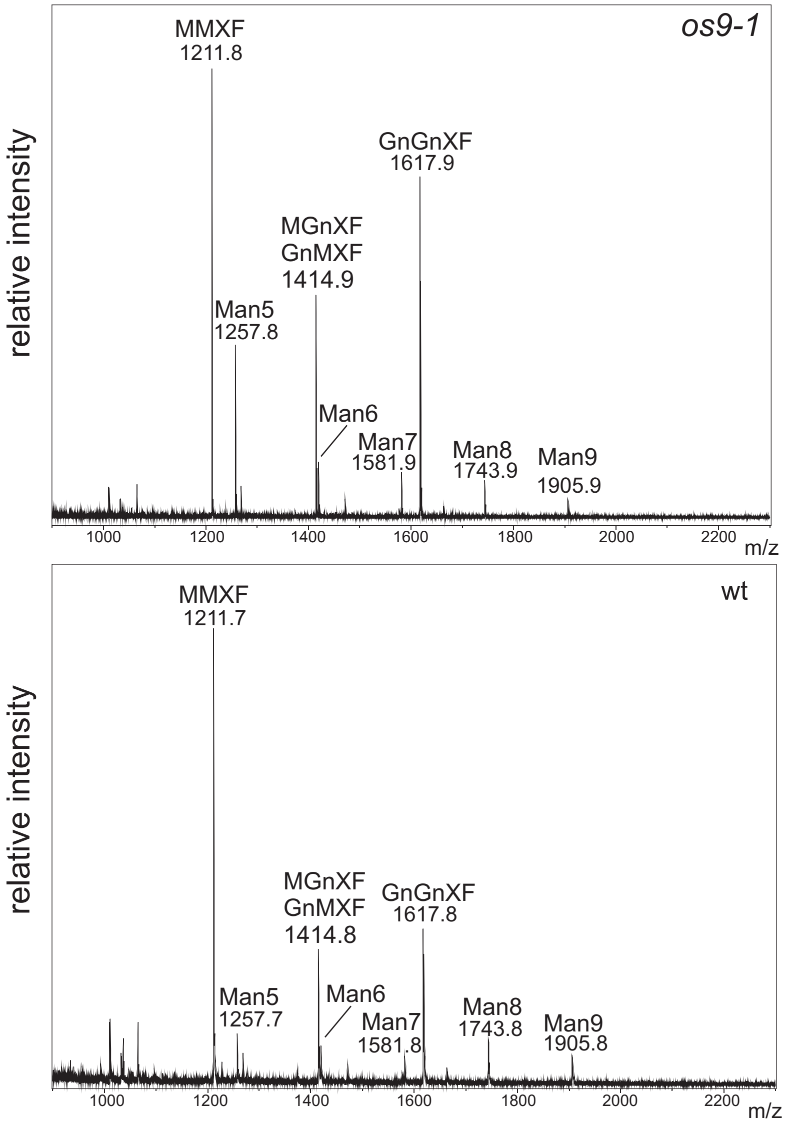

Fig. S2. Matrix-assisted laser desorption ionization time-of-flight mass spectrometry (MALDI-TOF-MS) of total N-glycans extracted from leaves of *A. thaliana* plants. Spectra of *os9-1* and wild-type plants are shown. The following oligosaccharide structures are indicated: MMXF: Man3XylFucGlcNAc2; Man5: Man5GlcNAc2; MGnXF/GnMXF: GlcNAcMan3XylFucGlcNAc2; Man6: Man6GlcNAc2; Man7: Man7GlcNAc2; GnGnXF: GlcNAc2Man3XylFucGlcNAc2; Man8: Man8GlcNAc2; Man9: Man9GlcNAc2.

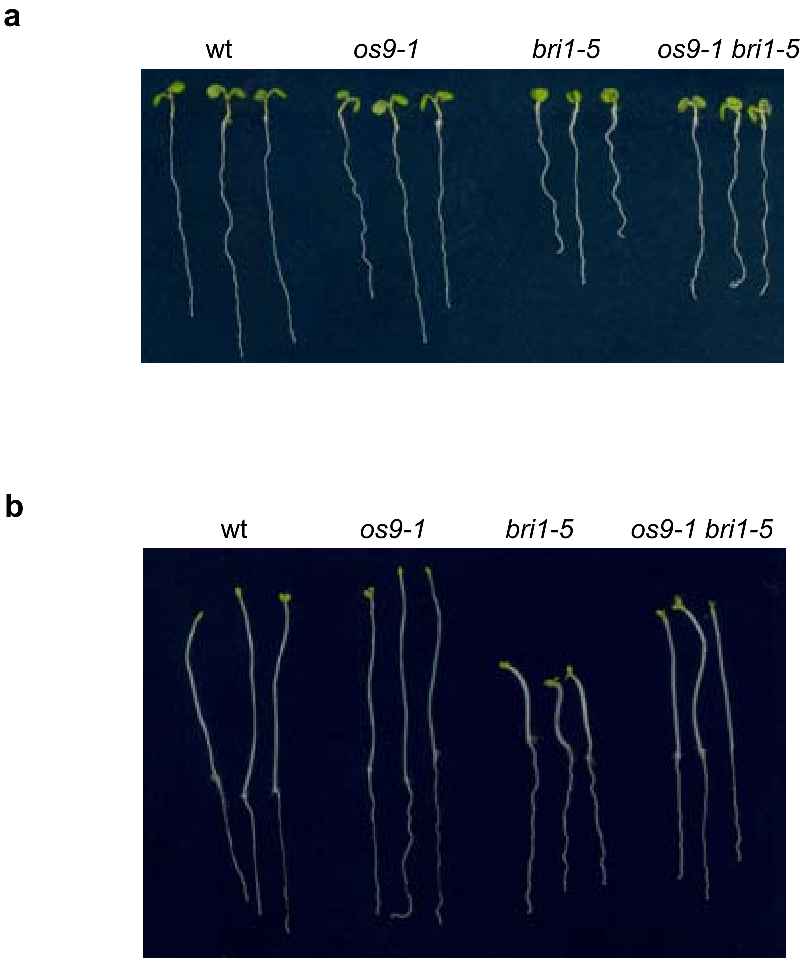

Fig. S3. *os9-1* suppresses the *bri1-5* phenotype. Growth of different plants on 1x MS medium with 1.5% sucrose for 8 days at 22°C **(a)** under long-day conditions (16h-light/8h-dark) or **(b)** in the dark.

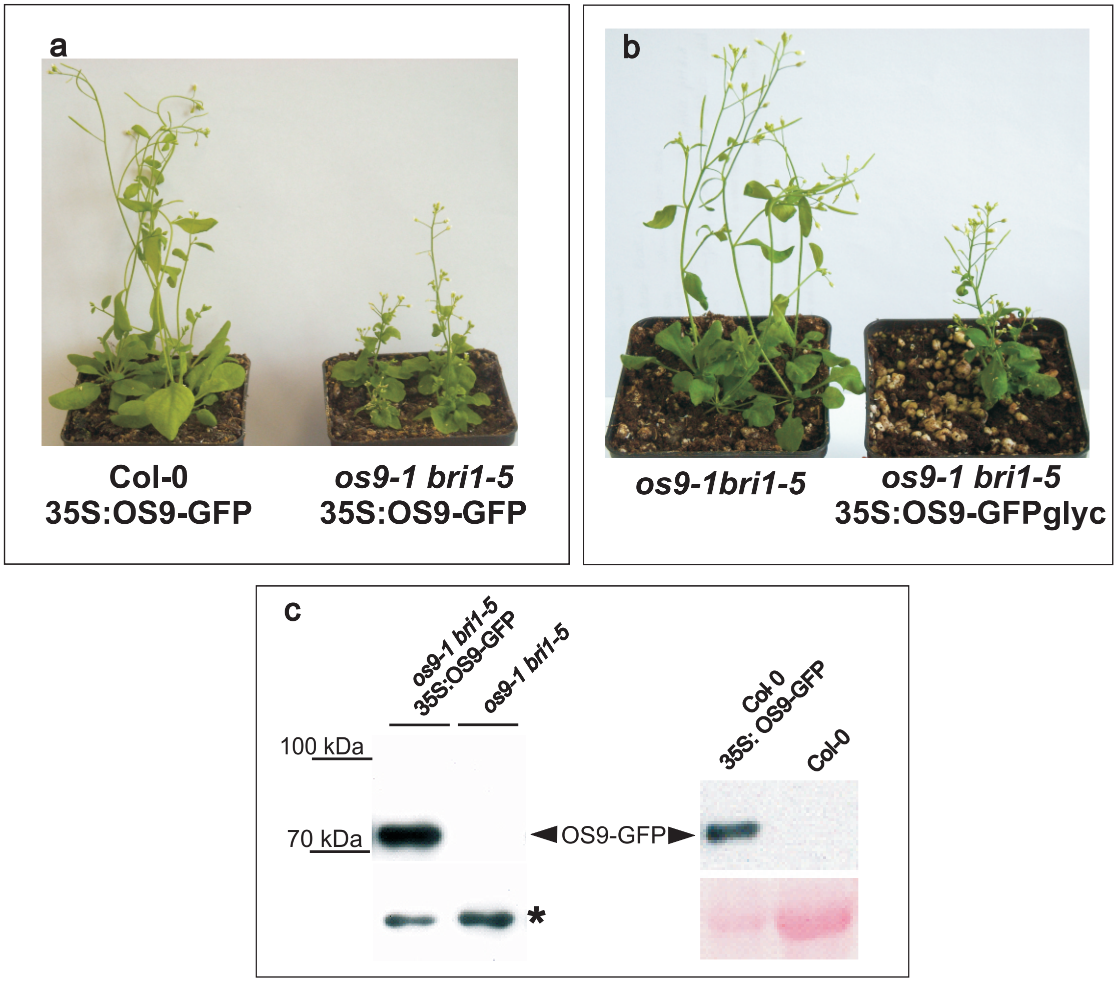

Fig. S4. Expression of OS9-GFP or OS9-GFPglyc in *os9-1 bri1-5* plants restores the *bri1-5* growth phenotype. *os9-1 bri1-5* double mutants were floral dipped with **(a)** 35S:OS9-GFP or **(b)** 35S:OS9-GFPglyc. **(c)** Positive transformants were analyzed by immunoblotting with anti-OS9 antibodies for the presence of OS9-GFP expression or OS9-GFPglyc. The asterisk on the immunoblot denotes an unspecific band that served as a loading control.

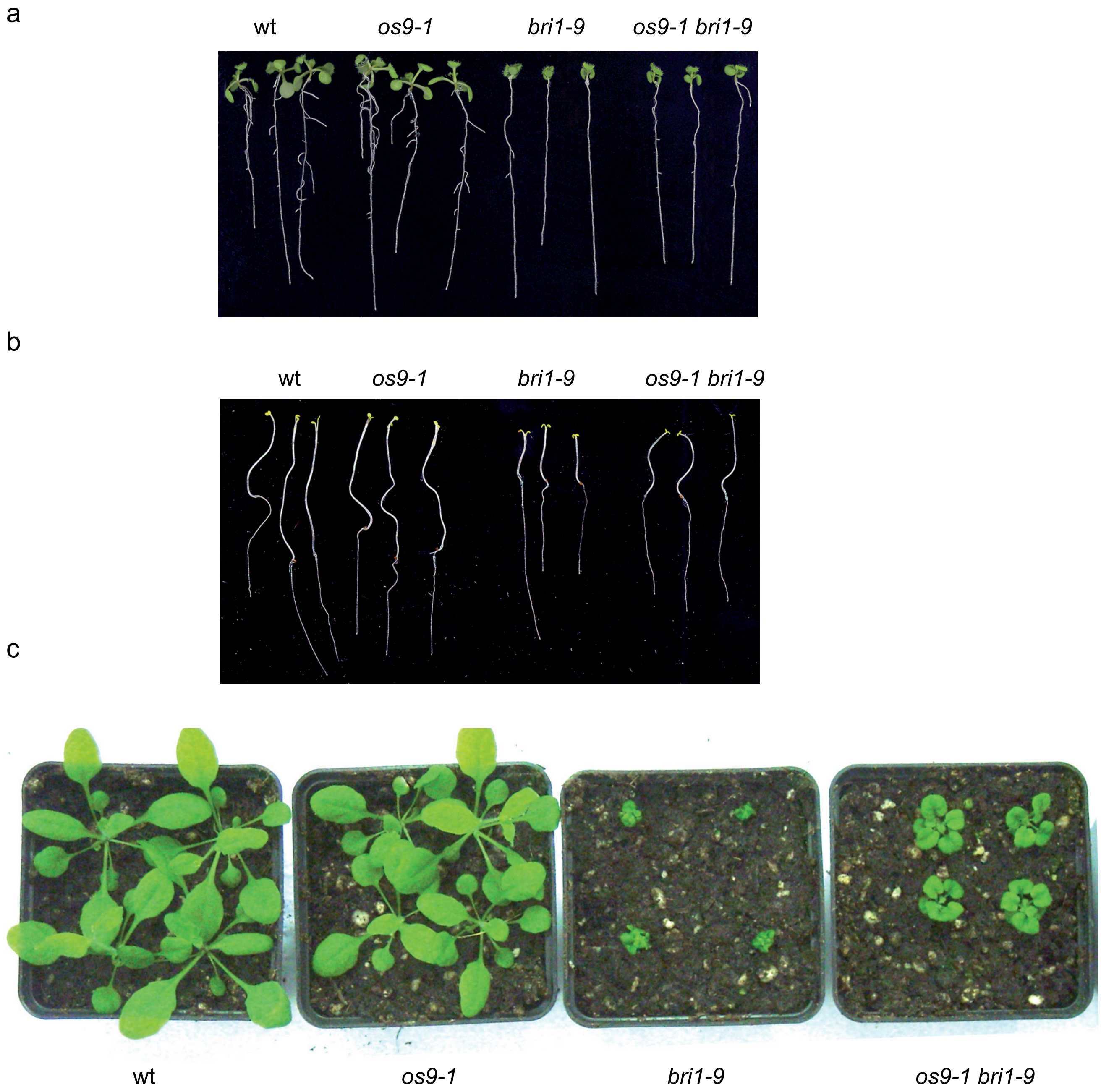

Fig. S5. *os9-1* partially suppresses the *bri1-9* phenotype. Growth of different plants on 1x MS medium with 1.5% sucrose for 2 weeks at 22°C **(a)** under long-day conditions (16h-light/8h-dark) or **(b)** in the dark. **(c)** Growth phenotype of 24 day-old soil-grown plants.

a

wt | *os9-1* | *bri1-6* | *os9-1 bri1-6*

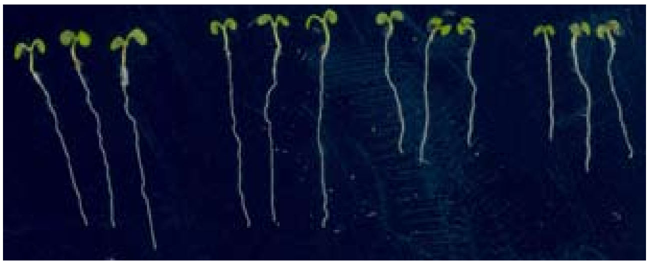

b

wt | *os9-1* | *bri1-6* | *os9-1 bri1-6*

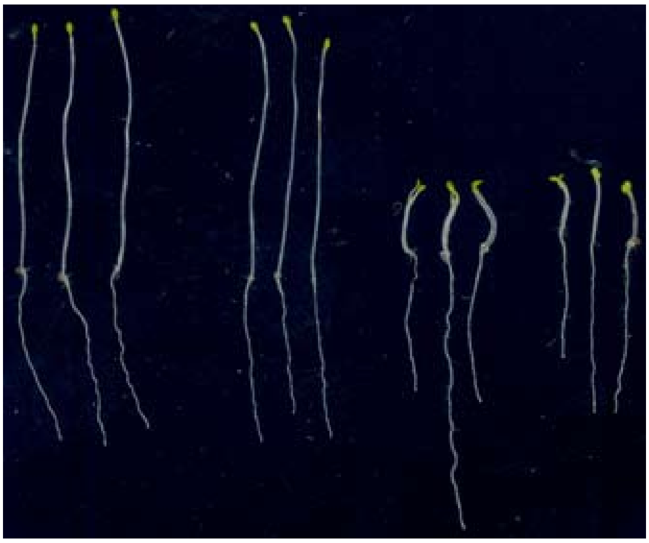

Fig. S6. *os9-1* does not suppress the *bri1-6* phenotype. Growth of different plants on 1x MS medium with 1.5% sucrose for 8 days at 22°C **(a)** under long-day conditions (16h-light/8h-dark) or **(b)** in the dark.

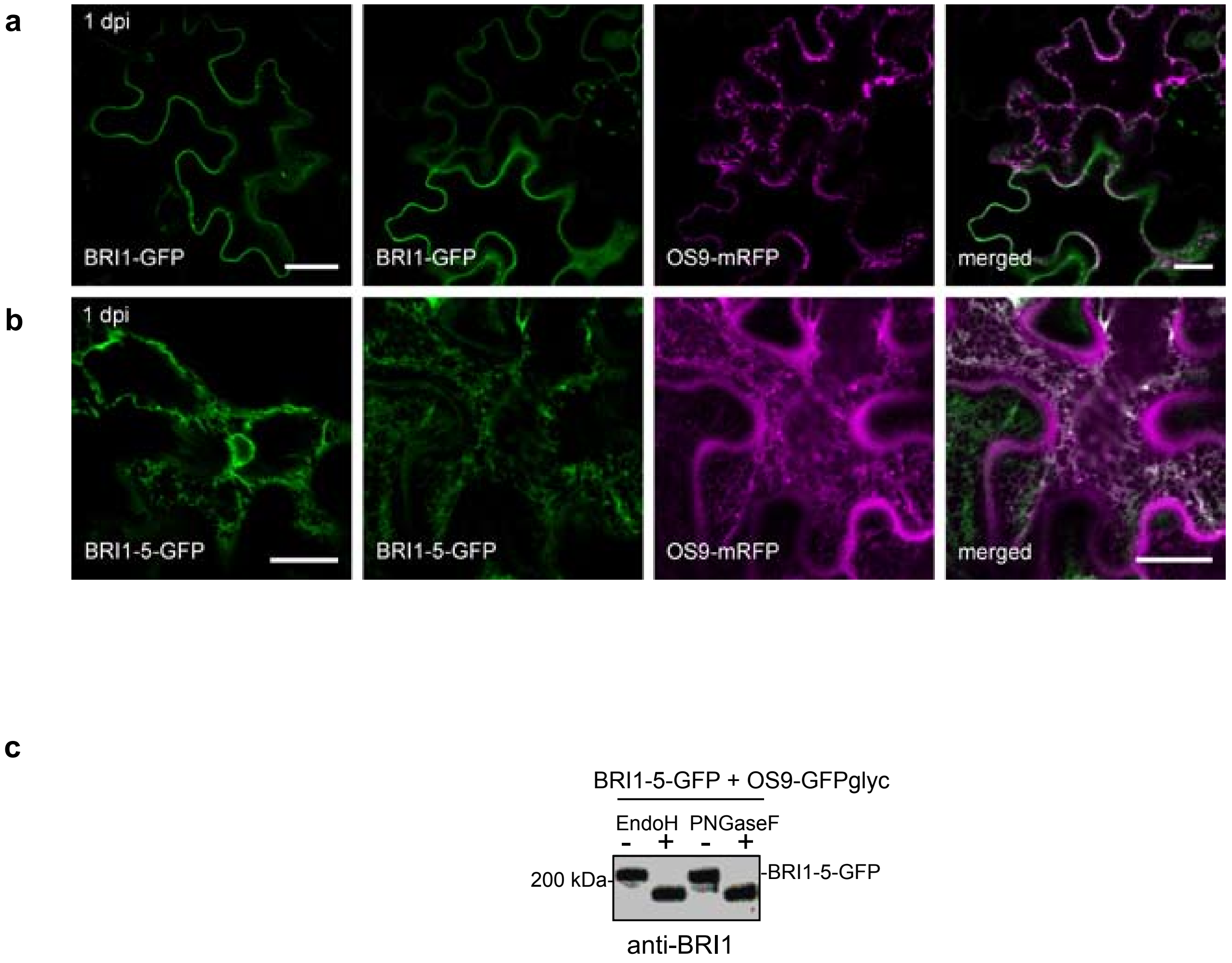

Fig. S7. BRI1-5-GFP is retained in the ER. Co-expression of different BRI1-forms in *N. benthamiana* leaf epidermal cells. Confocal images were taken 1 day after infiltration (OD_{600} = 0.15). Scale bars = 20 µm. **(a)** Co-expression of BRI1-GFP (in green) and OS9-mRFP (in magenta). The first image shows BRI1-GFP alone, followed by the images showing co-expression. **(b)** Co-expression of BRI1-5-GFP (in green) and OS9-mRFP (in magenta). The first image shows BRI1-5-GFP alone, followed by the images showing co-expression. **(c)** Immunoblot analysis suggests that BRI1-5 carries only oligomannosidic N-glycans indicative of ER-localization of BRI1-5 and OS9. Transient expression was done in *N. benthamiana* ΔXF plants, which contain low amounts of core α1,3-fucose. Complex N-glycans from this plant are therefore sensitive to PNGase F digestion.

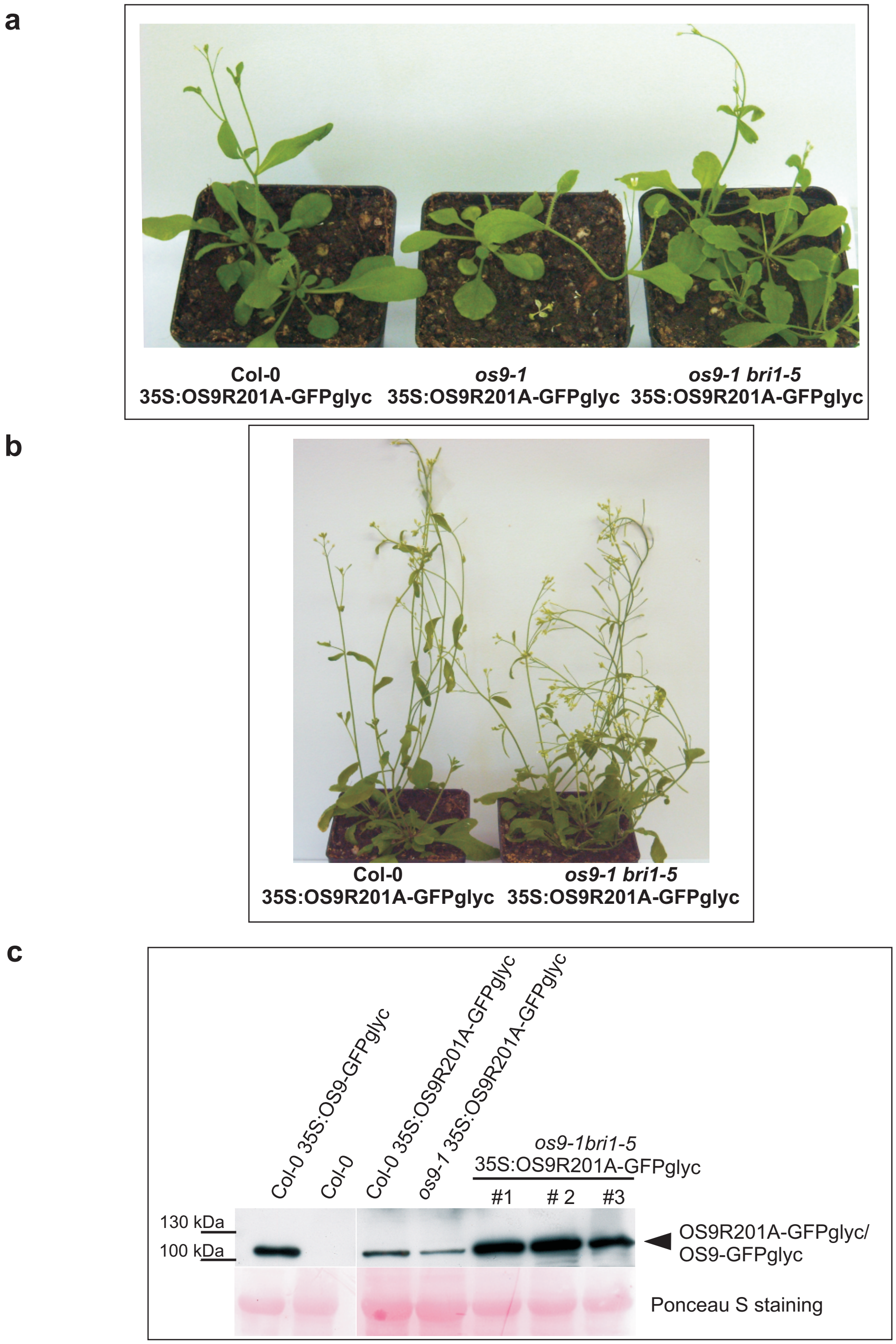

Fig. S8. Expression of OS9R201A-GFPglyc does not restore the *bri1-5* growth phenotype in *os9-1 bri1-5* plants. *os9-1 bri1-5* double mutants, *os9-1* mutants and wild-type (wt) were floral dipped with 35S:OS9R201A-GFPglyc. Positive transformants were grown on soil **(a** and **b)** and OS9R201A-GFPglyc expression was confirmed by **(c)** immunoblotting with anti-OS9 antibodies for the presence of OS9-GFPglyc expression.

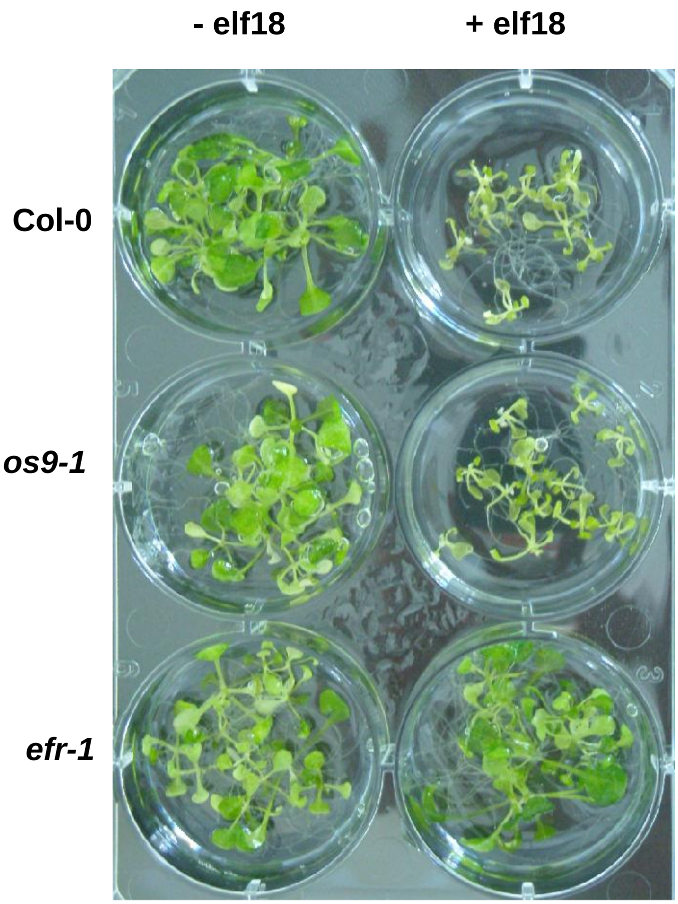

Fig. S9. *os9-1* seedlings are sensitive to elf18. A seedling growth inhibition assay in the absence (-elf18) or presence (+ elf18) of elf18 peptide is shown. The elf18 peptide blocks seedling growth of *os9-1* similar to wild-type seedlings (Col-0), while the mutant *efr-1* (Zipfel et al., 2006) that served as a control is insensitive to elf18. Seven-day-old seedlings were incubated for one week in 1x MS liquid medium containing 1% sucrose in the presence of 50 nM elf18 peptide.

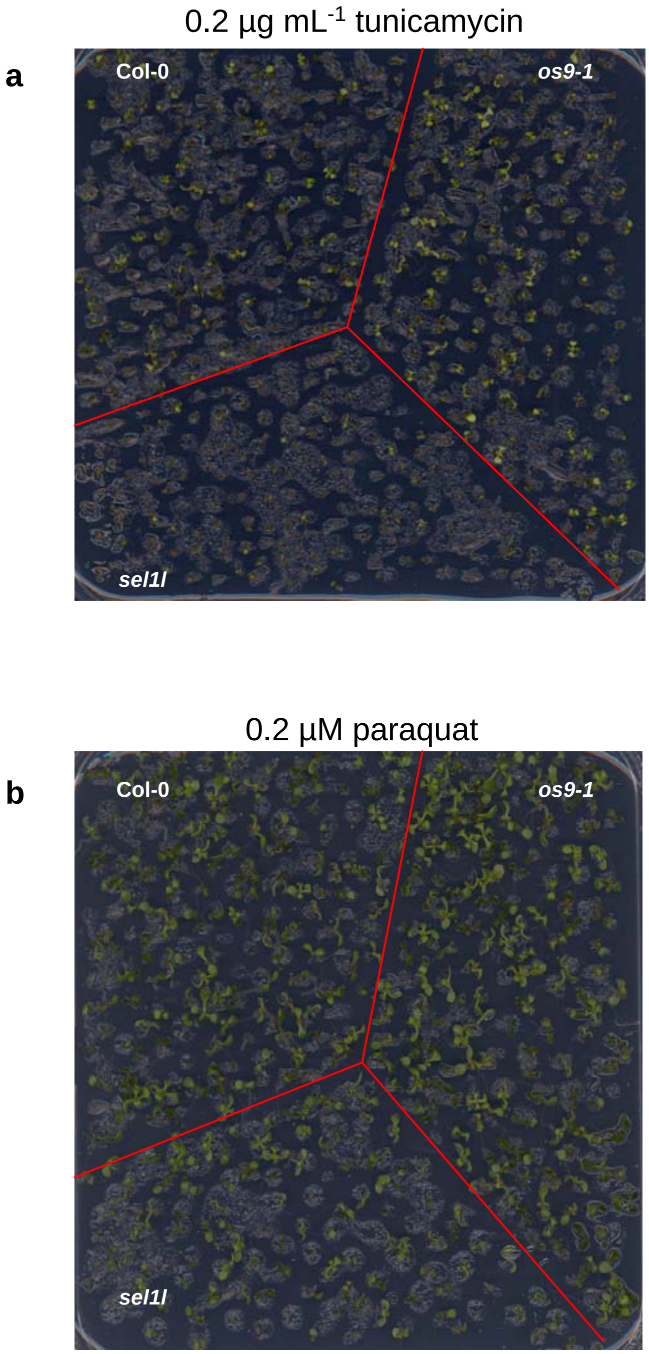

Fig. S10. Effect of tunicamycin and paraquat on *os9-1* seedlings. Seeds were germinated on ½x MS medium containing 1.5% sucrose **(a)** in the presence of 0.2 µg mL^{-1} tunicamycin or **(b)** in the presence of 0.2 µM paraquat. *sel1l* was used as a control (Liu et al., 2011). Seedlings were photographed after 11 days of treatment.
